# Global Burden of Leptospirosis: Estimated in Terms of Disability Adjusted Life Years

**DOI:** 10.1371/journal.pntd.0004122

**Published:** 2015-10-02

**Authors:** Paul R. Torgerson, José E. Hagan, Federico Costa, Juan Calcagno, Michael Kane, Martha S. Martinez-Silveira, Marga G. A. Goris, Claudia Stein, Albert I. Ko, Bernadette Abela-Ridder

**Affiliations:** 1 Section of Epidemiology, Vetsuisse Faculty, University of Zürich, Zürich, Switzerland; 2 Fundação Oswaldo Cruz, Ministério da Saúde, Salvador, Bahia, Brazil; 3 Department of Epidemiology of Microbial Diseases, Yale School of Public Health, New Haven, Connecticut, United States of America; 4 Institute of Collective Health, Federal University of Bahia (UFBA), Salvador, Brazil; 5 Center for Analytical Sciences, Yale School of Public Health, New Haven, Connecticut, United States of America; 6 WHO/FAO/OIE and National Leptospirosis Reference Center, Royal Tropical Institute, KIT Biomedical Research, Amsterdam, The Netherlands; 7 Division of Information, Evidence, Research and Innovation (DIR), World Health Organization Regional Office for Europe, Copenhagen, Denmark; 8 Department for the Control of Neglected Tropical Diseases, World Health Organization, Genève, Switzerland; University of Tennessee, UNITED STATES

## Abstract

**Background:**

Leptospirosis, a spirochaetal zoonosis, occurs in diverse epidemiological settings and affects vulnerable populations, such as rural subsistence farmers and urban slum dwellers. Although leptospirosis can cause life-threatening disease, there is no global burden of disease estimate in terms of Disability Adjusted Life Years (DALYs) available.

**Methodology/Principal Findings:**

We utilised the results of a parallel publication that reported global estimates of morbidity and mortality due to leptospirosis. We estimated Years of Life Lost (YLLs) from age and gender stratified mortality rates. Years of Life with Disability (YLDs) were developed from a simple disease model indicating likely sequelae. DALYs were estimated from the sum of YLLs and YLDs. The study suggested that globally approximately 2·90 million DALYs are lost per annum (UIs 1·25–4·54 million) from the approximately annual 1·03 million cases reported previously. Males are predominantly affected with an estimated 2·33 million DALYs (UIs 0·98–3·69) or approximately 80% of the total burden. For comparison, this is over 70% of the global burden of cholera estimated by GBD 2010. Tropical regions of South and South-east Asia, Western Pacific, Central and South America, and Africa had the highest estimated leptospirosis disease burden.

**Conclusions/Significance:**

Leptospirosis imparts a significant health burden worldwide, which approach or exceed those encountered for a number of other zoonotic and neglected tropical diseases. The study findings indicate that highest burden estimates occur in resource-poor tropical countries, which include regions of Africa where the burden of leptospirosis has been under-appreciated and possibly misallocated to other febrile illnesses such as malaria.

## Author Summary

Leptospirosis is a zoonotic bacterial disease that affects vulnerable populations such as rural subsistence farmers and urban slum dwellers. Although leptospirosis can cause life-threatening clinical manifestations such as pulmonary hemorrhage syndrome and has a worldwide distribution, to date, the global burden of leptospirosis has not been estimated. The estimated 1.03 million cases annually result in a total of approximately 2.90 million Disability Adjusted Life Years. For comparison, this is over 70% of the global burden of cholera. Most of this burden of leptospirosis is the result of premature death and is suffered disproportionately by young adult males in resource-poor tropical countries. These estimates place leptospirosis as a leading cause of disease burden amongst zoonotic agents.

## Introduction

Leptospirosis is a neglected emerging zoonotic disease that has an important public health impact worldwide, especially within economically vulnerable populations such as urban slums and rural subsistence farmers [1]. Although it can have high fatality and is recognised as the most common and widespread zoonotic disease, the worldwide burden of morbidity and mortality is still unknown [2–4]. Consequently, leptospirosis often is not considered a public health priority. Quantification of the burden of this neglected zoonotic disease will assist in facilitating informed dialogue about the relative importance of leptospirosis among other public health challenges, and guide priorities for resource allocation for surveillance, prevention and control, and research.

Leptospirosis predominantly causes a nonspecific febrile syndrome that is clinically difficult to distinguish from other causes of febrile illness [3,5,6]. Leptospirosis may have life-threatening manifestations that are responsible for most of the worldwide burden. These include acute renal and pulmonary failure and fulminant multi-system disease [7]. These forms occur in 5–15% of clinical leptospirosis cases, but case fatality of these forms may be 50% or higher [1,8,9]. Leptospirosis may be an important cause of undiagnosed febrile illness [5, 10–13]. However, HIV does not seem to be associated with more severe disease [5]. Furthermore, because most cases are reported in young adult males, it can have a substantial economic impact in low and middle income countries [7,14].

Disability Adjusted Life Years (DALY) is a health metric used by the World Health Organization (WHO) [15] and the Global Burden of Diseases Study [16] to estimate the burden of disease. One DALY is a health gap measure, equating to one year of healthy life lost. This metric allows direct comparison between public health problems in common terms that primarily reflect the impact on economic productive capacity. Table 1 gives a list of the abbreviations used in this manuscript.

**Table 1:** List of abbreviations.

AFR*	WHO African Region
AMR*	WHO Region of the Americas
DALY	Disability Adjusted Life Year
DW	Disability weight
EMR*	WHO Eastern Mediterranean Region
EUR*	WHO European Region
GBD	Global Burden of Disease
LERG	Leptospirosis Epidemiology Reference Group of the WHO
SEAR*	WHO South-East Asian Region
SPSH	Severe Pulmonary Hemorrhage Syndrome
UI	Uncertainty Interval
WHO	World Health Organization
WPR*	WHO Western pacific region
YLD	Years Lived with Disability
YLL	Years of Life Lost

* WHO regions are further subdivided into subregions. **A:** very low child, very low adult mortality; **B** Low child, low adult mortality; **C**: Low child, high adult mortality; **D**: High child, high adult mortality; **E**: High child, very high adult mortality. List of countries in each subregion is given in S1 File.

The Leptospirosis Epidemiology Reference Group (LERG) [17] of the WHO was established to estimate the global impact of leptospirosis both in terms of morbidity and mortality and in terms of DALYs. LERG therefore undertook a systematic review of the worldwide literature and developed a modeling strategy due to the sparsity of available data to achieve these aims. This review included predominantly hospital-based surveillance studies, which capture more severe forms leading to hospitalization, but do not measure mild febrile forms that form the base of the iceberg of leptospirosis cases. From the data generated it was estimated that there are approximately 1.03 million cases globally each year resulting in 58,900 deaths [18]. These mortality and morbidity estimates have been used in this report to estimate the global burden in terms of Disability Adjusted Life Years (DALYs).

## Methods

### DALY calculation

DALYs result from the sum of the number of years of life lost due to mortality (YLLs) and the number of years lived with a disability (YLDs) due to the disease [19].

The formulas for the YLLs, YLDs and DALYs calculations are described below:

YLLs=N X L

where N is the number of deaths per year and L is the standard life expectancy at age of death in years.

YLDs=I X DW X L

where I is the number of incident cases per year, DW is the disability weight and L the average duration of the disease until remission or death in years. This incidence based approach was the method undertaken in the present study.

Alternatively YLDs can be calculated as

YLDs=P X DW

where P is the point prevalence estimate of the disease in terms of total cases at that time point. There is little material difference between the two methods for estimating YLDs, except for diseases of chronic sequelae where the incidence or prevalence is changing over time.

DALYs=YLLs+YLDs

This is essentially the simplest formulation of the DALY calculation which was used for estimating the latest global burden of disease estimate [20]. It is also possible to age weight the estimate where years lost at certain times of life are weighted more heavily, placing a premium on years lost by young adults. Discounting can also be used to estimate future losses at present day values. Neither age weighting nor discounting were undertaken in this study as in GBD 2010 [20].

### Mortality Due to Leptospirosis and YLLs

The incidence and mortality data used to estimate the global burden of leptospirosis was reported by Costa et al. [17] in which fatality from acute leptospirosis was estimated at 6.95%. The number of deaths by age and gender calculated with the aid of population census data was used to estimate the numbers of YLLs. The life table used to estimate life expectancy at age of death was the same life table used in GBD 2010 [20]. The 2010 fractional population within each age and gender group was obtained from the UN population division [21]. Further YLLs were also estimated derived from foetal losses following miscarriage in pregnant women who suffer acute leptospirosis. One retrospective cohort study [22] and one review [23] were identified which reported acute leptospirosis in a total of 26 pregnant women. Of these, 14 suffered foetal loss (54%). The incidence in pregnant women was estimated from the number of births in the reference year and the incidence in women in the strata of reproductive age (15 years to 50 years). The proportion of pregnant women suffering from leptospirosis who suffered foetal loss was assumed to be a mean of 54%. Each foetal death was assumed to lose 86 YLL, the life expectancy at birth using the GBD 2010 life table [20]. This approach to foetal death is consistent with other burden estimates such as that of congenital toxoplasmosis [24,25]. This life expectancy is the average mean achievable life expectancy regardless of cultural, health or socioeconomic factors. Therefore is standardized so that each individual has the same potential years of life at birth. A society where life expectancy at birth that is lower than this reflects the likely burden of disease or injury that occurs in that society.

### Disease Sequelae and Disability Weights

Leptospirosis is an acute disease with a high mortality rate among hospitalized cases. Classically, this is a biphasic disease, with a non specific febrile leptospiraemic phase that may be followed by more severe manifestations during a second immune phase, including acute multiorgan dysfunction sometimes requiring extensive treatment in intensive care settings [2]. Most morbidity in acute leptospirosis is due to renal and pulmonary failure. According to a report by LERG summarizing a systematic review of literature on leptospirosis sequelae [26], acute renal injury is reported in 36% of patients (range, 0–88%), carrying fatality of 12% (range, 0–62%). Acute lung injury was reported in 17% of cases (range, 0 to 62%), with fatality of 25% (range, 2–87%); severe pulmonary hemorrhage syndrome is observed in 10% of patients, with case fatality exceeding 50% [8,9]. Chronic sequelae from severe leptospirosis are less well described, however these may include extreme fatigue, myalgia, malaise, and headache, with durations that can persist beyond two years after discharge [27]. Neurological and psychological complications stemming from extended intensive care unit treatment may also occur. We defined chronic sequelae as likely to be lasting longer than 2 months.

**Fig 1:**
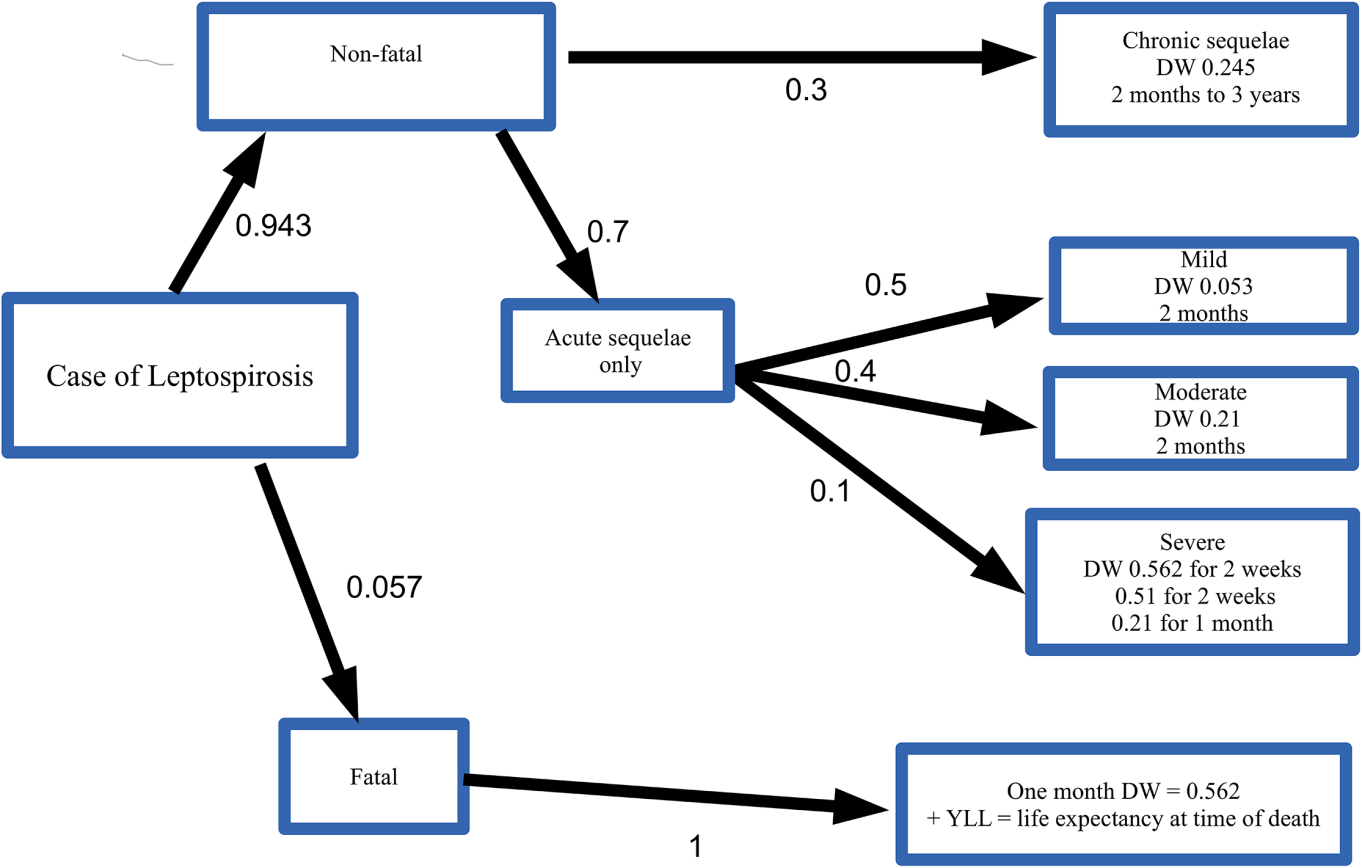
Decision tree to assign probabilities and DWs for sequelae of leptospirosis.

These clinical syndromes were used to develop a disease model to assign disability weights (Fig 1). All fatal cases were assumed to have severe disease that would require dialysis before death. GBD 2010 gives the DW for dialysis of 0·573 [28] (SE = 0·088) of duration of 1 month. Non-fatal cases were given disability weights and durations reflective on the acute nature of the disease and the spectrum of clinical signs. A total of 10% of non-fatal cases were given a DW for dialysis for 1 month followed by a DW of 0·21(SE = 0·04) for severe infection for a further 1 month. For 40% of the non-fatal cases, a DW of 0·21 for a duration of 2 months was given. The remaining 50% of non-fatal cases were given a DW of mean 0·053 (SE = 0·012) for 2 months. Acute lung injury is not one of the unique health states which has a disability weight defined in GBD 2010 [28]. Although this sequela occurs in approximately 17% of cases [25], it is of short duration and therefore likely adequately captured for severe infection.

Uveitis is also frequently reported as a sequel to acute leptospirosis. A systematic search of the literature identified one prospective cohort study [29] which determined the incidence in patients treated for leptospirosis. A total of 174 cases were followed for 30 months. Of these 32 developed eye disease during follow up, with 21 suffering from anterior uveitis, with just 6 (3·4%) developing visual symptoms. Therefore a mean of 3·4% of non-fatal cases were assigned a DW of 0·004 (SE = 0·002), the DW of mild impairment of distance vision in GBD 2010 [28], for a duration of 1 month.

Long-term sequelae due to leptospirosis are known to occur, but not extensively described in the literature. A systematic search of the literature only revealed one manuscript that reported long term sequelae [27]. In this report, active case finding identified 225 individuals previously diagnosed with acute leptospirosis. Of these 68 (30·2%) reported chronic symptomatology, of which 57 indicated the duration of the symptomatology (Table 2). Therefore a mean of 30·2% of individuals who had non-fatal leptospirosis were given a DW of 0·245 (SE = 0·04), the DW for infectious disease, post-acute consequences [28]. The duration of symptomatology was estimated from the data in Table 2. For example a mean of 17·5% had symptomatology lasting 4–6 months whilst a mean of 21·1% had symptoms lasting greater than 24 months. In this study the mean duration of acute symptomatology, as defined by period of hospitalization and duration of symptoms prior to hospitalization was 16 days.

**Table 2:** The duration of chronic sequelae reported by 57 individuals following acute leptospirosis.

Duration in months	n	%
<2	11	19.3
2–4	7	12.3
4–6	10	17.5
6–8	5	8.8
8–10	2	3.5
10–12	6	10.5
12–18	3	5.3
18–24	1	1.8
>24	12	21.1

### Calculation of Uncertainty Intervals

We incorporated uncertainty surrounding the incidence and mortality rates and disability weights into the analysis using Monte-Carlo techniques. Model estimates of mortality and incidence with their respective standard errors were obtained from Costa et al [18]. Lognormal distributions were constructed around these estimates using the SE of the estimates. Likewise DW were reported in GBD 2010 [28] with their standard errors and hence these were used to construct normal distributions around these estimates. The proportion of cases that had chronic symptoms was modeled using a beta distribution with alpha and beta parameters of 69 and 158, reflecting the sample size of those reporting chronic sequelae in Goris et al [27]. The duration of long term sequelae was modeled using a Dirichlet distribution (the multivariate generalisation of the beta distribution) using a vector of parameters derived from the observed data reported in Table 2. The proportion of cases suffering mild vision loss was modeled with a beta distribution with alpha and beta parameters of 7 and 168. The proportion of pregnant women with leptospirosis suffering from foetal loss was modeled using a beta distribution with alpha and beta parameters of 15 and 13 respectively. Monte-Carlo samples of the incidence, mortality, proportion with sequelae and DWs were taken and used to estimate the DALY. This was repeated 10,000 times. The mean and 2.5 and 97.5 percentiles of the resulting Monte-Carlo distribution was used to report the uncertainty estimates of the DALYs, YLLs and YLDs.

### Sensitivity Analysis

Because of the uncertainties surrounding the disease model for those recovering from leptospirosis, the short duration of the disease sensitivity analysis was undertaken to estimate the effect on varying the disease weight for leptospirosis on the total DALY estimates. This consisted of estimating the contribution of YLDs to the estimate by recalculating the DALYs of two extreme assumptions. The first gave a DW of leptospirosis of zero during the first 2 months following infection. The alternative was giving a DW of leptospirosis a maximum of 1 for a duration of 2 months.

## Results

**Fig 2:**
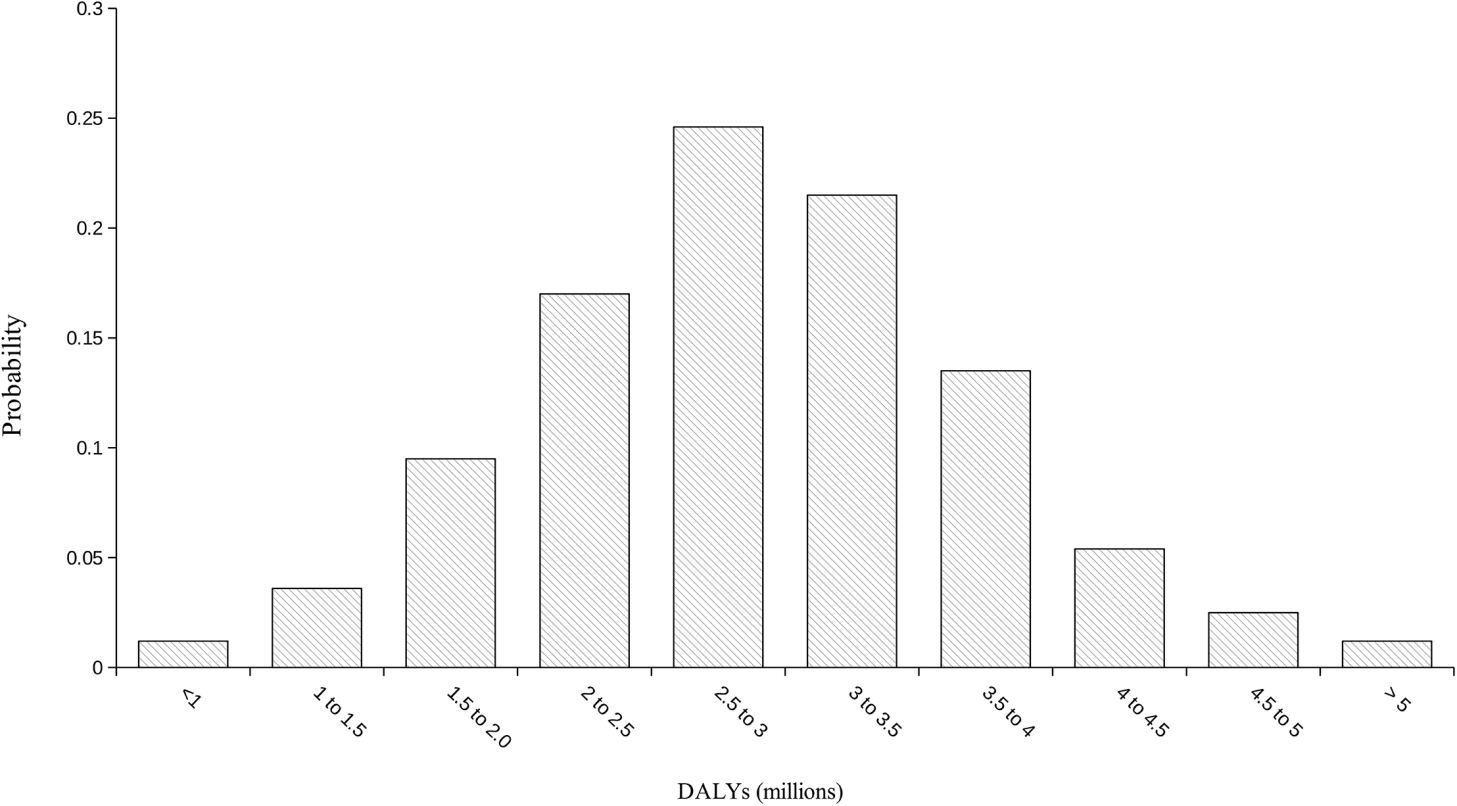
Frequency distribution of the global burden of leptospirosis.

**Fig 3:**
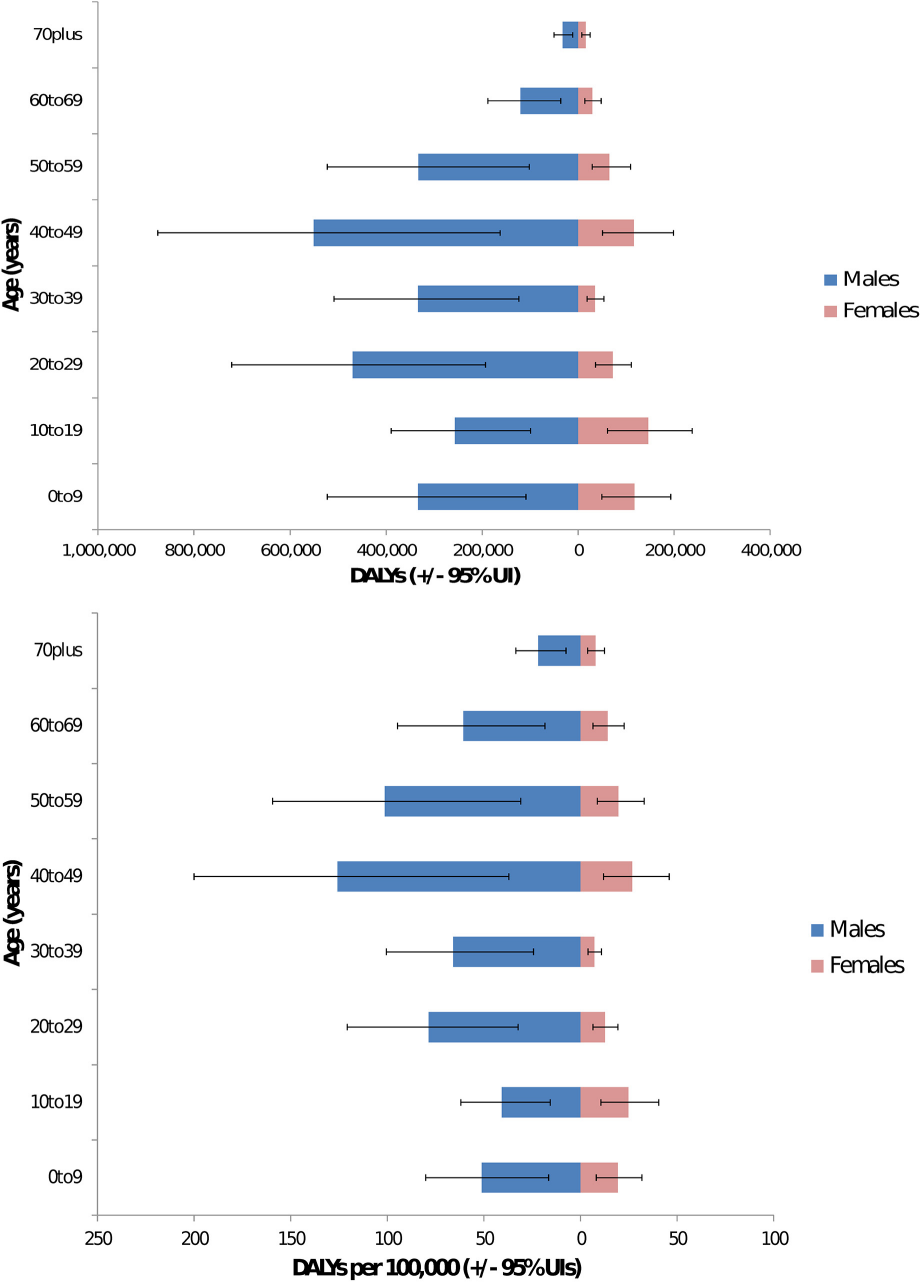
Proportion of burden by age and gender: Top—total burden; bottom—DALYs per 100,000. The latter controls for population size of each age group.

**Fig 4:**
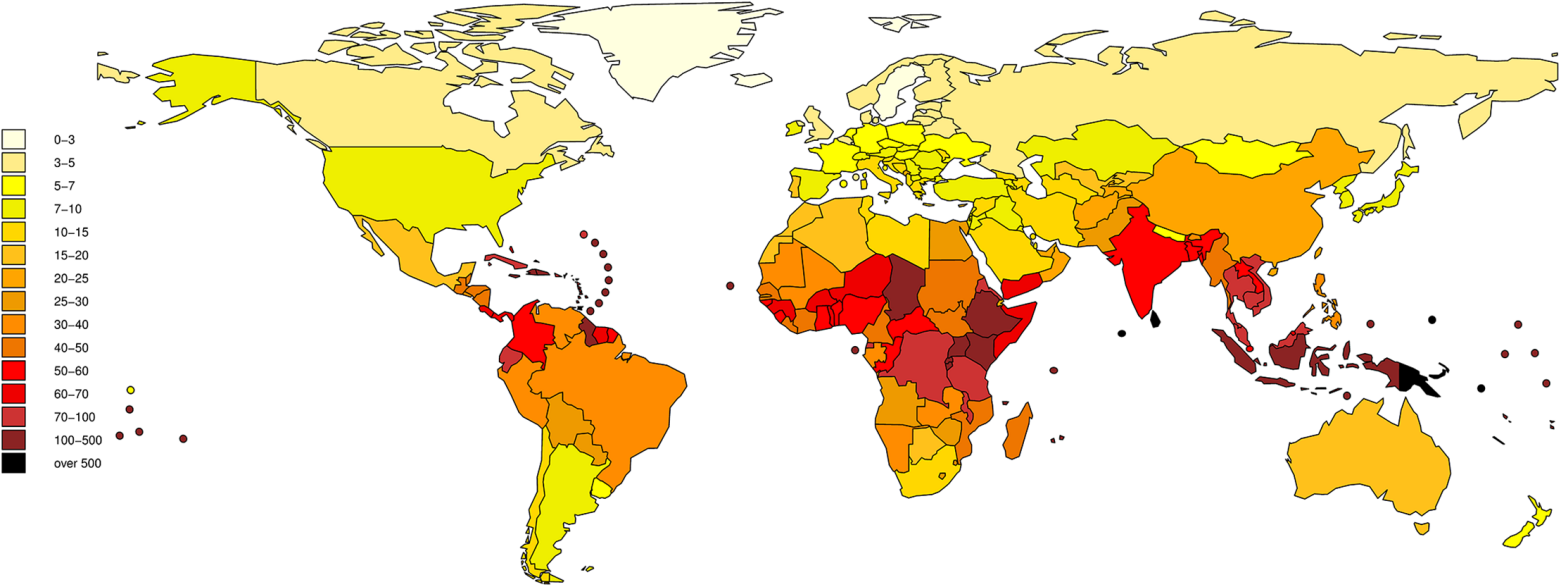
Burden of leptospirosis in terms of DALYs/100,000 per year.

The global burden of leptospirosis was estimated at 2·90 million DALYs per annum (UIs 1·25–4·54 million). This consisted of 2·80 million YLLs (1·16 million– 4·46 million) and 103,200 (38,800–188,100) YLDs. This represents an incidence of 41·8 DALYs per 100,000 population per year (UI 18·1–65·5). The frequency distribution of the global DALY estimate is illustrated in Fig 2. Males were predominantly affected with an estimated 2·33 million DALYs (0·95–3·66 million) or approximately 80% of the total burden. Young adults aged 20–49 had an estimated burden of 1·5 million DALYs (0·65–2·32 million) or approximately 52% of the total. Of these young men age 20–49 have a burden of 1·30 million (0·56–2·03 million) or 45% of the total burden. The proportion of the burden of leptospirosis by gender and age is illustrated in Fig 3. Results are given by WHO and GBD region in Tables 3 and 4 respectively. The global burden in terms of DALYs per 100,000 is illustrated in Fig 4. Individual country estimates can be found in the on line supporting information (S2 File). Sensitivity analysis indicated that DWs had very little influence on the estimates for the global burden of disease. Thus the YLLs resulted in 96·4% of the DALYs. This would be the DALY estimate of the burden if the DW was set at zero for all sequelae. When the DW is set at the highest possible of 1 during the acute phase for all cases, the burden is estimated at 3·03 million DALYs (UIs 1·40–4·71 million), of which YLDs are 248,200 (83,800–417,300). In this scenario, the YLLs still represent 91·8% of the total burden. In addition because the uncertainty intervals of the central estimate includes the two extreme estimates by a wide margin, it illustrates that choice of DW has little influence on the burden estimate. Likewise foetal losses suffered by pregnant women affected by leptospirosis resulted in approximately 12,200 YLLs (UIs 8200–16700), representing just 0·4% of total YLLs. The YLDs contributed by leptospirosis associated uveitis was a mean of just 14 and thus make a trivial contribution to the burden of disease.

**Table 3:** Annual burden of disease due to leptospirosis by WHO region.

WHO Region	YLLs (UIs)	YLDs (UIs)	DALYs (UIs)	DALYs/100,000 (UIs)
AFR D	173868 (76570–279087)	3804 (1098–6741)	177672 (81221–279691)	45 (21–71)
AFR E	426603 (175431–693252)	9533 (2704–17746)	436136(192221–702083)	97 (43–156)
AMR A	38142 (14341–63600)	2024 (634–3906)	40167 (15812–67238)	11 (4.3–18.2)
AMR B	151923 (55265–249188)	7487(2377–14103)	19410 (63734–256190)	33 (13–52)
AMR D	43639 (16061–75569)	1924(626–3787)	45562 (17920–77300)	54 (21–92)
EMR B	20444 (7576–32718)	1005 (321–1843)	21450 (8551–33534)	13 (5.0–20)
EMR D	120872 (46430–196605)	4127 (1319–7557)	125000 (50368–202035)	74 (30–119)
EUR A	28002 (10461–45545)	1733 (529–3243)	29736 (12272–47467)	6.9 (2.8–11)
EUR B	21979 (8493–35552)	1059 (335–1963)	23038 (9568–36687)	10 (4.1–15.9)
EUR C	9029 (3303–15783)	358(110–694)	9387 (3633–16057)	4.1 (1.6–6.9)
SEAR B	441910 (160102–738270)	18264 (5748–36001)	460174 (179180–754509)	143 (56–234)
SEAR D	761833 (277855–1327117)	28941 (8641–57986)	790775 (308846–1356193)	53 (21–91)
WPR A	17384 (6325–29203)	1105 (360–2122)	18488 (7112–30168)	12 (4.5–19)
WPR B	499226 (197169–822826)	24168 (8070–45659)	523393 (219260–844190)	31 (11–52)

**Table 4:** Annual burden of disease by GBD region. Country level estimates are available in the S2 File.

GBD region	YLLs (UIs)	YLDs (UIs)	DALYs (UIs)	DALYs/100,000 (UIs)
High Income Asia Pacific	23954 (9463–39400)	1514 (455–2822)	25469 (11008–42030)	12 (5.2–20)
Central Asia	10438 (4019–17189)	421 (148–779)	10860 (4403–17580)	14 (5–22)
East Asia	289746 (112946–500221)	14262 (4459–27943)	304008 (120122–529149)	22 (8.7–38)
South Asia	794710 (309100–1337935)	29363 (8569–55874)	824073 (353505–1388078)	50 (18–88)
South East Asia	632799 (252538–1048433)	26749 (8253–49167)	659548 (278381–1077471)	137 (58–224)
Australasia	3919 (1421–6982)	239 (78–462)	4158 (1640–7156)	16 (6.4–28)
Caribbean	53697 (23318–86957)	2107 (663–3897)	55804 (22863–89131)	127 (52–203)
Central Europe	9699 (4273–159499)	493 (158–890)	10191 (4210–16687)	8.5 (3.5–14)
Eastern Europe	7491 (2688–13463)	293 (88–552)	7784 (3264–13613)	3.8 (1.6–6.6)
Western Europe	27832 (11521–45203)	1654 (538–3022)	29486 (13665–45178)	7.1 (3.2–11)
Andean Latin America	23483 (10045–40563)	1180 (382–40563)	24663 (9775–43105)	46 (18–81)
Central Latin America	71594 (28958–119632)	3639 (1123–6923)	71659 (31841–118669)	33 (13–54)
Southern Latin America	4658 (1817–7835)	242 (70–460)	4901 (2225–8197)	8.0 (3.7–13)
Tropical Latin America	59480 (22848–110022)	2727 (808–5586)	62207 (24247–110181)	31 (12–54)
North Africa/Middle East	77597 (30981–125979)	3352 (1035–5923)	80950 (33807–126649)	18 (7.4–28)
High Income North America	24211 (9284–41987)	1331 (418–2611)	25542 (10530–44224)	7.3 (3.0–13)
Oceania	55412 (21071–99855)	1728 (481–3529)	57140 (21807–97645)	515 (196–879)
Central Sub-Saharan Africa	75334(27995–130292)	1275 (357–2456)	76610 (29464–129234)	78(30–133)
Eastern Sub-Saharan Africa	378563 (148923–598337)	9216 (3277–17693)	365599 (151309–593478)	106 (42–168)
Southern Sub-Saharan Africa	12471 (4787–19987)	242 (81–464)	12713 (5250–19901)	18 (7.4–28)
Western Sub-Saharan Africa	157870 (60039–247295)	3240 (1115–6048)	154316 (53063–255603)	48 (18–75)

## Discussion

To date leptospirosis has not been specifically included in any of the GBD studies. Therefore the present results fill an important knowledge gap that directly contributed to the neglected status of leptospirosis by presenting an informed prioritization of this disease among other public health research, prevention, and control priorities. We show that leptospirosis is an important infectious disease worldwide, with a global burden of approximate 2·90 million DALYs per year, most of which occurs among low and middle-income tropical countries. Although GBD 2010 used prevalence based YLDs which will underestimate the burden of chronic diseases where there is increasing populations, comparisons of the burden of leptospirosis with diseases that have a major impact on low income countries can be made. Table 5 illustrates the burden per 100,000 of leptospirosis compared to the estimates of other neglected and tropical diseases estimated by GBD 2010 [16]. Thus the burden of leptospirosis appears to be of a similar magnitude to that of schistosomiasis, leishmaniasis and lymphatic filariasis and about 73% of that of cholera. Furthermore, 2.90 million DALYs represent the equivalent of all the inhabitants of a city the size of Rome or Nairobi losing one year of healthy life.

**Table 5:** The burden of leptopirosis per 100,000 compared to GBD estimates for the burden of various tropical and neglected diseases.

Diseases	DALYs per 100,000 [16].
Malaria	1200 (921–1594)
Cholera	65 (49–84)
Leishmaniasis	48 (32–71)
Schistosomiasis	48 (25–91)
Leptospirosis	42 (18·1–66)
Lymphatic filariasis	40 (26–58)
Rabies	21 (12–39)
	52 (22–145)*

*A higher burden of rabies was estimated by Hampson et al. [30], due to variations in methodology and estimates of mortality rates.

Leptospirosis also predominantly affects males (80% of the total burden), and young adults (52% of the total burden affects adults aged 20–49). Leptospirosis is therefore a disease that causes substantial economic burden, especially in lower and middle income countries for which young adult males are the most important demographic for economic productivity. Our estimates arise directly from the mortality and morbidity estimates of leptospirosis reported elsewhere [18], and any uncertainties will parallel those reported. These include sparsely distributed morbidity and incidence data which required the use of modelling techniques to estimate missing data. There was also incomplete laboratory testing of suspect cases. Furthermore, the majority of the incidence data was obtained from hospital based surveillance studies which likely underestimate the morbidity of leptospirosis. Similarly there were likely underestimates of mortality. Additional levels of uncertainty will be in the development of DWs from the disease model, and any inaccuracies that may have arisen from assigning fatal cases to the correct age stratum. DWs contribute to the YLD part of the DALY and not to the YLL. Therefore, As YLDs contribute a small amount to the DALYs due to leptospirosis, uncertainties in DWs have little effect on estimates of the total disease burden. In contrast, inaccuracies in the age at death of fatal cases could make a more substantial contribution into uncertainties surrounding the YLLs. In common with the report in incidence and mortality, the uncertainty intervals are wide with a 95% confidence that the burden is between 1·16 million and 4·46 million DALYs. In addition this study further confirms that the areas with the highest impact include Oceania, the Caribbean, parts of Latin America, Sub-Saharan Africa, East Asia, and parts of South-East Asia (Fig 4).

Despite an extensive search of the literature, only one paper was found which had adequate data to use for modeling chronic sequelae. The report [27] was essentially a retrospective cohort study on patients identified through active case finding. Data from 225 patients was analyzed of which 68 reported chronic sequelae. The sequelae reported included depression, extreme fatigue, headache and malaise, myalgia, joint pains, back pain, shoulder pain, stomach pain, vertigo and tinitus. These symptoms appeared consistent with the infectious disease: post-acute consequences (fatigue, emotional lability, insomnia) reported in GBD 2010 [28] and hence these chronic sequelae were assigned a DW of 0.254. However, this report was in the context of an upper income country with a high standard of medical case. In a low income setting it may be an underestimate of the frequency and duration of chronic sequelae.

For evidence of foetal loss during pregnancy there were a number of individual case reports, but only two reports of case series or retrospective cohort studies could be found that were able to estimate the probability of abortion following infection during pregnancy [22,23]. From these two studies 14 of 26 pregnant women, confirmed to be suffering from acute leptospirosis, suffered an abortion. Although a small sample size, it was possible to introduce this data into the model and also model the uncertainty surrounding the estimated fetal lethality rate due to leptospirosis during pregnancy. However despite the apparent high risk of foetal loss, the contribution to the burden of disease is low. This is because the incidence of disease in women is much lower than men.

Because of the problems of diagnosis and data gaps, the true burden could be considerably higher. Fever is a commonly presenting complaint among persons seeking health care in low-resource areas. In many areas, malaria is over-diagnosed, and patients without malaria have poor outcomes. For example in a recent study in northern Tanzania 870 individuals with febrile illnesses were investigated. The clinical diagnosis in 60.7% of these was malaria, but the actual frequency of malaria was just 1.6%. Leptospirosis was identified in 8.8% [11]. Similarly a study in the Amazon basin indicated that leptospirosis was a more frequent cause of acute febrile illness than malaria [31]. Consequently it is quite possible that the burden of leptospirosis is considerably higher, with substantial numbers of DALYs misallocated to other infectious diseases such as malaria.

In our estimates, we used incidence based YLDs. In contrast GBD 2010 in general used prevalence based estimates for the YLDs[18]. For acute diseases of short duration there is no practical difference between the two methods. In addition, YLDs make a very minor contribution to the burden estimate of leptospirosis as individuals who survive the disease tend to make a full recovery after a relatively short time period with few if any long term sequelae. This is confirmed by the sensitivity analysis where extreme DW of 0 or 1 only had a very small influence on the DALY total. Thus the DALY is essentially driven by the YLLs. This also indicates that the accurate mortality estimates far outweigh morbidity estimates in estimating the DALY. Thus to improve on these estimates studies should be targeted to obtain accurate estimates of mortality due to leptospirosis, and importantly the age of death.

We did not use any form of social weighting based on age in the calculation of DALYs, which in the case of leptospirosis would have led to a higher estimate for the global burden, as the highest burden occurs in young productive adults. Our study demonstrates that the burden of leptospirosis is of a similar order of magnitude as several other neglected tropical diseases such as leishmaniasis, lymphatic filariasis and hookworm disease (see data in Murray et al [16]). This burden falls disproportionately on young males between 20 and 49 years of age, in low income tropical countries, the disease is likely to have a substantial economic impact.

### Disclaimer

The findings and conclusions of this report are those of the authors and do not necessarily represent the official views, decisions or policies of the World Health Organization

## Supporting Information

S1 File Glossary of definitions and WHO subregions. (DOC)

S2 File Country level burden estimates of leptospirosis by WHO and GBD region. (XLS)
