# Supplementary material for: Global Burden of Leptospirosis: Estimated in Terms of Disability Adjusted Life Years
Source: PLoS Negl Trop Dis. 2015 Oct 2;9(10):e0004122. doi: 10.1371/journal.pntd.0004122 (PMC4591975; doi:10.1371/journal.pntd.0004122)
Supplement: S1 File — (DOC) [file pntd.0004122.s001.doc]

**GLOSSARY**

*Burden of disease*

In the context of this Initiative, the term “burden of disease” follows the principles of the Global Burden of Disease Study, and includes the quantification of morbidity, all disabling complications and mortality in a single summary measure (DALY).

*DALY (disability-adjusted life year)*

A health gap measure that combines the years of life lost due to premature death (YLL) and the years lived with disability (YLD) from a disease or condition, for varying degrees of severity, making time itself the common metric for death and disability. One DALY equates to one year of healthy life lost.

WORLD HEALTH ORGANIZATION REGIONS

For subregions:

**A:** very low child, very low adult mortality; **B** Low child, low adult mortality; **C**: Low child, high adult mortality; **D**: High child, high adult mortality; **E**: High child, very high adult mortality

For epidemiological reasons, overseas territories of European countries and non recognized territories have been included in the most appropriate WHO region.

**WHO African Region:**

**AFR D**

Algeria, Angola, Benin, Burkina Faso, Cameroon, Cape Verde, Chad, Comoros, Equatorial Guinea, Gabon, Gambia, Ghana, Guinea, Guinea-Bissau, Liberia, Madagascar, Mali, Mauritania, Mauritius, Niger, Nigeria, Sao Tome and Principe, Senegal, Seychelles, Sierra Leone, Togo.

Also includes: Mayotte and Reunion

**AFR E**

Botswana, Burundi, Central African Republic,Congo, Côte d’Ivoire, Democratic Republic of the Congo, Eritrea, Ethiopia,Kenya, Lesotho, Malawi, Mozambique, Namibia, Rwanda, South Africa, Swaziland, Uganda, United Republic of Tanzania, Zambia, Zimbabwe.

**WHO Region of the Americas:**

**AMR A**

Canada, Cuba, United States of America.

Also includes: American Virgin islands, Bermuda, British Virgin islands, Cayman Islands, French Guinea, French West Indies, Netherlands' Antilles, Puerto Rico & St Pierre and Miquelion

**AMR B**

Antigua and Barbuda, Argentina, Bahamas, Barbados, Belize,Brazil, Chile, Colombia, Costa Rica, Dominica, Dominican Republic, El Salvador, Grenada, Guyana, Honduras, Jamaica, Mexico, Panama, Paraguay, Peru, Saint Kitts and Nevis, Saint Lucia, Saint Vincent and the Grenadines, Suriname, Trinidad and Tobago, Uruguay, Venezuela.

Also Includes: Anguilla, Guadeloupe, Monserat & Turks and Caicos

**AMR D**

Bolivia, Ecuador, Guatemala, Haiti, Nicaragua,

**WHO South-East Asia Region:**

**SEAR B**

Indonesia, Sri Lanka, Thailand

**SEAR D**

Bangladesh, Bhutan, Democratic People’s Republic of Korea, India, Maldives, Myanmar, Nepal, Timor-Leste.

**WHO European Region:**

**EUR A**

Andorra, Austria, Belgium, Croatia, Cyprus, Czech Republic, Denmark, Finland, France, Germany, Greece, Iceland, Ireland, Israel, Italy, Luxembourg, Malta, Monaco, Montenegro, Netherlands, Norway, Portugal, San Marino, Serbia, Slovenia, Spain, Sweden, Switzerland, United Kingdom.

Does not include overseas territories which for epidemiological reasons have been included in more appropriate WHO regions

**EUR B**

Albania, Armenia, Azerbaijan, Bosnia and Herzegovina, Bulgaria, Georgia, Kyrgyzstan, Poland, Romania, Slovakia,Tajikistan, The former Yugoslav Republic of Macedonia, Turkey, Turkmenistan, Uzbekistan.

**EUR C**

Belarus, Estonia, Hungary, Kazakhstan, Latvia, Lithuania, Republic of Moldova, Russian Federation, Ukraine.

**WHO Eastern Mediterranean Region:**

**EMR B**

Bahrain, Iran, Jordan, Kuwait, Lebanon, Libyan Arab Jamahiriya, Oman, Qatar, Saudi Arabia, Syrian Arab Republic, Tunisia, United Arab Emirates

Also includes: Palastinian Territories

**EMR D**

Afghanistan, Djibouti, Egypt, Iraq,, Morocco,Pakistan, Somalia, South Sudan, Sudan, Yemen.

Also includes: Western Sahara

**WHO Western Pacific Region:**

**WPR A**

Australia, Brunei Darussalam, Japan, New Zealand, Singapore.

**WPR B**

Cambodia, China, Cook Islands, Fiji, Kiribati, Lao People’s Democratic Republic, Malaysia, Marshall Islands, Micronesia, Mongolia, Nauru, Niue, Palau, Papua New Guinea, Philippines, Republic of Korea, Samoa,, Solomon Islands, Tonga, Tuvalu, Vanuatu, Viet Nam.

Also includes: French Polynesia and New Caledonia
